# Gender Differences in the Association between Cholesteryl Esters Transfer Protein Polymorphism (rs708272) and Plasma Lipid Levels in Hyperlipidaemic Participants at Hospital Universiti Sains Malaysia

**DOI:** 10.21315/mjms2023.30.2.9

**Published:** 2023-04-18

**Authors:** Amirul Faez Shamsudin, Nur Salwani Bakar

**Affiliations:** School of Health Sciences, Universiti Sains Malaysia, Kelantan, Malaysia

**Keywords:** statin, hyperlipidaemia, cholesteryl esters transfer protein, genetic polymorphism, low-density lipoprotein cholesterol

## Abstract

**Background:**

Single nucleotide polymorphism (SNP) in the *cholesteryl esters transfer protein* (*CETP*) gene (rs708272) was reported to affect statin efficacy. This study investigated the association between *CETP* rs708272 and statin’s lipid-lowering effects in hyperlipidaemic participants at Hospital Universiti Sains Malaysia, Kelantan.

**Methods:**

A total of 229 hyperlipidaemic statin users (96.1% Malays) were recruited, and a single blood sample (3 mL) was obtained for DNA extraction. The genotypes were determined using PCR-RFLP method and validated by sequencing analysis.

**Results:**

The minor allele frequency for rs708272 in all participants was 0.391, with no difference between females and males. At the baseline, the SNP was associated with different low-density lipoprotein (LDL-c) and triglyceride (TG) levels in females, but not males, when the GG and GA+AA genotypes were compared using a dominant genetic model. Regardless of the genotype, the total cholesterol and LDL-c levels decreased significantly (*P* < 0.001) in both genders after statin treatment, but the TG levels decreased exclusively in females with the GG genotypes. In both genders, high density lipoprotein levels were unaffected before and after the statin treatment.

**Conclusion:**

To improve the management of hyperlipidaemia, future research should consider patient gender when assessing the *CETP* rs708272 impact on LDL-c and TG.

## Introduction

Statin, also known as 3-hydroxy-3-methylglutaryl coenzyme A (HMG-CoA) reductase inhibitor, is the first line of therapy against hyperlipidaemia (1). Statin inhibits the conversion of HMG-CoA into mevalonate, which is a rate-limiting step in the cholesterol biosynthesis and finally lowers the plasma cholesterol levels (1). Statin was evident to reduce low density lipoprotein cholesterol (LDL-c) levels, a well-established risk factor for cardiovascular disease, by 24%–60% (2). According to a meta-analysis from 14 randomised trials, statin decreased the mortality rate of coronary heart disease (CHD) by 19% (3). It is worth to note that statin efficacy in lowering LDL-c can vary due to a variety of factors, including genetic polymorphisms (4).

The gene transcribing cholesteryl esters transfer protein (CETP), located at chromosome 16 is regarded as one of the most important genes related to statin efficacy. This gene plays a key role in maintaining cholesterol homeostasis by participating in reverse cholesterol transport (RCT) (5). It promotes the transfer of cholesteryl ester from high density lipoprotein cholesterol (HDL-c) to apolipoprotein B (apo-B)-containing particles in exchange for triglycerides (TG), thereby reducing the concentration of HDL-c and increasing non-HDL-c (6). CETP also transfers the HDL-cholesteryl ester to very low-density lipoprotein (VLDL) and LDL-c, and this LDL-c will be then taken up mainly by the liver through LDL receptors; therefore, this pathway is termed indirect RCT (7). Therefore, CETP can have either a pro-atherogenic or anti-atherogenic function in cholesterol metabolism depending on the LDL receptor integrity (7). If the LDL receptor is defective, the transfer of esterified cholesterol to apo-B containing particles may result in cholesterol build-up and atherosclerosis (7). CETP is anti-atherogenic when the LDL receptor is functioning well since it promotes cholesterol uptake into hepatocytes, thus preventing atherosclerosis (7). Because of its important functions in lipid metabolism, polymorphisms in CETP gene have been extensively studied for their associations with lipid profiles, especially HDL-c level across different populations (8–10).

The most commonly studied polymorphism in the CETP gene is Taq-1 ß polymorphism (i.e. rs708272), which is a single nucleotide polymorphism (SNP) that results in a base substitution at nucleotide 277th position (G277A) in intron 1 (11). The common allele G of the *CETP* rs708272 is associated with low HDL-c levels and thus, higher index of atherogenicity (10). Minor allele A, on the other hand, is associated with decreased serum CETP concentrations, higher HDL-c levels and a lower risk of CHD (10, 12). This association, however, remains controversial since it varies across populations presumably owing to sample size variations and other contributing factors, such as body mass index (BMI), alcohol consumption and gender (13). Previous population-based studies have also found that the common allele G of the SNP was significantly linked to an increased risk of atherogenic dyslipidaemia (13–15). However, there was insufficient evidence linking the common allele G of the SNP to a higher index of atherogenicity, leading to a debate whether the lipid changes were caused by the statin or the SNP. Furthermore, the findings in a Thai population recently confirmed that the allele G carriers of the SNP were associated with higher susceptibility to atherogenic dyslipidaemia due to decreased HDL-c level, but this effect was somewhat altered in patients receiving statin (16). Considering the demographic profile, such as gender in relation to lipid changes, there is a controversial debate about which gender group was most likely to be affected by the SNP. A case-control study with 556 coronary artery disease (CAD) patients and 414 controls in Chinese population found that the minor allele carriers in male had significantly higher HDL-c levels than the homozygous dominant of the SNP (*P* = 0.041), but no significant association between lipid levels and *CETP* rs708272 was found in females (17). An earlier study in Caucasian subjects (*n* = 526) found that the AA genotype of the *CETP* rs708272 in females was significantly associated with the highest level of HDL-c when compared to other genotypes (*P* < 0.001 for the trend) (18).

Because the CETP gene affects lipid parameters, the *CETP* rs708272 is likely to affect statin efficacy in lipid-lowering. To date, no studies have been conducted in Malaysia on the SNP among statin users. Therefore, we intend to look into the relationship between *CETP* rs708272 and statin efficacy, and their effects on lipid levels, as well as to provide preliminary data for statin pharmacogenetics in a subset of Malaysian population.

## Methods

### Participant Recruitment and Data Collection

This study was a retrospective cross-sectional study that included 229 hyperlipidaemic statin users between the ages of 18 years old to 70 years old from February 2018 to September 2020. All participants were outpatient statin users in *Klinik Rawatan Keluarga* (KRK), Hospital Universiti Sains Malaysia (HUSM), Kelantan. The inclusion criteria included being on statin therapy for at least 6 weeks (the majority of whom had been diagnosed with hyperlipidaemia and hypertension) and adhering to the recommended statin therapy as determined and monitored by a clinician. Hyperlipidaemia refers to an increase in concentration of one or more plasma or serum lipids i.e. LDL-c > 2.6 mmol/L, TG > 1.7 mmol/L and HDL-c < 1.1 mmol/L. Hypertension was also diagnosed among the hyperlipidaemic participants if the systolic blood pressure was > 130 mmHg or the diastolic blood pressure was > 80 mmHg on each of two successive readings obtained by the clinician. Participants who were taking other lipid-lowering agents or medications that interacted with statin were excluded from the study. Participants were also confirmed to be free of diseases like chronic kidney disease, thyroid disorders, uncontrolled diabetes mellitus (HbA1c above 7.0 %) and familial hypercholesterolaemia.

The participants who came to the KRK for their routine lipid follow-up check-up were invited to take part in this study. Before the recruitment process, a written consent form has been obtained from all participants. The participant’s information was obtained from an online hospital database in the HUSM according to the date they initially started the statin treatment (baseline). Depending on their follow-up dates, serial participants’ lipid profiles were recorded from the same clinical database.

### Biochemical Parameter Analysis

For the lipid profile tests, approximately 2 mL of venous blood was extracted from each participant after an overnight fast (9 h–12 h). The biochemical parameters, including total cholesterol (TC), TG, HDL-c and LDL-c were analysed by an enzymatic colorimetric method using Hitachi 912 autoanalyzer (RANDOX laboratories, United Kingdom) available in the department of Chemical Pathology of HUSM.

### DNA Extraction

Following the participants’ recruitment, 3 mL of venous blood was withdrawn from each participant and stored in K_2_ EDTA tubes for genotyping. DNA extraction was performed using GeneAll Blood ExGene SV Blood mini kit (GeneAll Biotechnology, Korea) and their DNA were kept in the final dilution buffer at −20°C until further use. The quality and quantity of the extracted DNA were determined using Infinite M200 microplate reader (Tecan).

### Genotyping Using PCR-RFLP

The CETP rs708272 was genotyped using polymerase chain reaction-restriction fragment length polymorphism (PCR-RFLP) method according to Ordovas et al. (13). Taq-1α, the restriction enzyme used, recognised the dominant allele G and cut the PCR product to produce 174 bp and 361 bp fragments. Recessive allele produced a 535 bp of uncut fragment. To ensure the quality control of the PCR-RFLP genotyping results, 10% out of total samples were randomly chosen and sent to the Human Identification Unit DNA (HID) Universiti Sains Malaysia for sequencing analysis. The sequencing results (refer Appendix 1) were confirmed by BLAST in the NCBI database (GenBank accession number: AY422211).

### Sample Size Calculation

The calculation of the sample size was based on the proportion comparison from two independent groups (19). Since there was no priori information regarding the effects of the SNP on lipid profiles in Malaysian population, we considered LDL-c reduction in GG+GA genotypes and AA genotypes as 0.35 (proportion in control, P0) and 0.23 (proportion in case, P1), respectively (20). To achieve the significance level (α) of 0.05 and the statistical power (1-β) at 80%, we needed at least 224 participants in total, without considering the dropout rate. The calculation was performed with the aid of an online calculator 4.0. (https://wnarifin.github.io/ssc_web.html).

### Statistical Analysis

SPSS software version 26.0 (IBM, United States) was used to perform statistical analysis for all clinical variables (expressed as mean, SD and 95% CI). The normality of each continuous variable was tested by histogram and box plots and finally confirmed by Kolmogorov-Smirnov test. For categorical variables, Pearson’s chi-squared test was used unless ≥ 20% of the cells had a count of less than 5, in which case, the Fischer’s exact test was used. Moreover, the chi-squared goodness of fit was used to determine whether the observed genotypic frequency was likely to follow those predicted by Hardy-Weinberg equilibrium (HWE) (*P* > 0.05). For continuous variables, *t*-test was used and in the case of non-parametric, Mann-Whitney test was used. If the normality assumption was met, the difference in mean scores for continuous variables was assessed using repeated measure ANOVA, and in the case of non-parametric, the Friedman test was used. Due to the multiple group comparisons, the Bonferroni correction was used to determine the statistically significant differences between the group means. The analysis for lipid parameters was further stratified based on the participant’s gender and *CETP* rs708272 genotypes to assess the association between gender, the SNP and lipid profiles. A dominant model (i.e. comparison between GG and GA + AA genotypes) was applied due to the homozygous mutants being rare. A *P*-value of ≤ 0.05 was considered statistically significant.

## Results

### Characteristics of the Study Participants

The information about participant’s demographic profiles, their diagnosis, the concurrent medications and the lipid profiles is shown in Table 1. Referring to the baseline lipid profiles, all the TC and LDL-c levels exceeded the normal ranges, the TG levels were borderline optimal, and the HDL-c levels were below the normal range. Despite being below the targeted normal range (> 1.5 mmol/L), the female participants had significantly (*P* < 0.001) higher HDL-c level at the baseline, but not other types of lipids, as compared to the male group (Table 1).

### Genotype Frequencies

**Figure 1:**
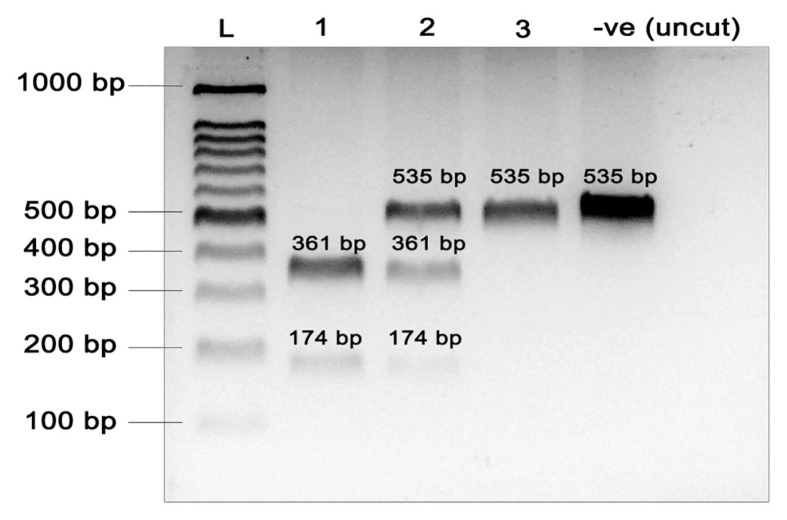
Restriction digestion of *CETP* rs708272 amplified products with restriction enzyme Taq-1α and visualised on 2% agarose gel; L = 100 bp molecular weight marker, Lane 1 = homozygous dominant (GG), Lane 2 = heterozygous (GA), Lane 3 = homozygous recessive (AA); −ve (uncut) = negative control (uncut PCR product)

Figure 1 indicates the presentation of bands using the restriction enzyme digestion for the *CETP* rs708272. The bands of the SNP matched to that produced by Ordovas et al. (13), with resulting fragments being 174 bp and 361 bp for the G allele and 535 bp for the uncut A allele. Table 2 summarises the genotypic frequencies and minor allele frequency (MAF) obtained from the current study and in comparison with the established healthy cohort data from the Ensemble (http://asia.ensembl.org/). Since no MAF data of the SNP among Southeast Asians were available in the database, we obtained the data from gene candidate studies available from the local studies to present the current status of the SNP allele frequencies. In this study, heterozygous genotypes (GA) were the most abundant (44.1%), followed by homozygous dominant (GG) and recessive genotypes (AA) with 38.9 % and 17.0 %, respectively. The genotypic frequency for the SNP in this study was in HWE (*P* = 0.736). There was no significant difference in the genotypic frequencies between Malaysian and East Asians, Vietnamese and Europeans. The genotypic frequencies of Malaysian population differed significantly from those in other populations, such as Thai (*P* < 0.001), Singaporean (*P* = 0.006) and African (*P* < 0.001) populations (Table 2).

Table 3 shows the statistical analysis between genotypes in *CETP* rs708272 and lipid levels using a dominant model. Comparing GG and GA+AA genotypes in all participants for their LDL-c level at the baseline, we found significant difference in the mean LDL-c level (*P* = 0.032) and a borderline significant difference for the mean TG level (*P* = 0.052). The participant stratification based on gender was carried out. The minor allele A carriers in females, but not in males, had significantly higher LDL-c (*P* = 0.007) and lower TG (*P* = 0.044) level at the baseline. The baseline levels of TC and HDL-c appeared to be unaffected by the SNP in the overall and gender-based analysis. After the statin treatment, all participants had significant (*P* < 0.001) decrease in TC and LDL-c levels. However, the HDL-c level was unaffected by the statin treatment. Significant (*P* < 0.001) TG decrease was observed in GG genotypes only within the overall participants and female group.

## Discussion

While statins are beneficial for lowering the LDL-c levels in the majority of hyperlipidaemic patients, some individuals do not effectively respond to statin treatment (4). The elevated blood LDL-c levels, in particular, have been identified as risk factors for cardiovascular diseases (CVD) (26). According to National Education Cholesterol Programme Adult Treatment Panel III, the LDL-c level is the major treatment target to reduce CVD events or death (2). In fact, based on the achievement of LDL-c therapeutic target (i.e. reduction below than 100 mg/dL), a 10-year risk of a major coronary event in the patients can be predicted (2). Depending on the statin types, the presence of gene variants has been shown to affect the degree of achieving the therapeutic lipid target (27). Statin pharmacological treatment is often recommended if one is unable to achieve LDL-c target level via non-pharmacological therapy (2, 26, 27).

Numerous previous studies linked the CETP polymorphism to an increased risk of atherogenic dyslipidaemia, and thus, CVD. However, this relationship lacks consistency due to sex specificity (15, 17). CETP modifies the lipid levels by mediating the inverse transfer of cholesteryl esters from HDL-c to atherogenic lipoproteins, resulting in a decrease in HDL-c levels (7). A SNP polymorphism in the CETP, such as rs708272, has been linked to an increased risk of CVD and changes in lipid levels (10, 13, 28, 29). To our knowledge, this is the first study to investigate the prevalence of *CETP* rs708272 polymorphism in a subset of hyperlipidaemic statin users in Malaysia, and to assess its impact on their lipid profiles.

MAF, a measure of the relative frequency of minor alleles of a SNP, is widely used in gene association studies because it allows researchers to distinguish common and rare variants in a population. We found consistent result for the MAF value of *CETP* rs708272 obtained in the present study (MAF = 0.391) among Malays, the majority ethnic group among the participants, and other populations (10, 13, 17, 21, 27). The MAF obtained in this study matched (*P* > 0.05) those found in the healthy subjects among East Asians, Europeans and Americans, but not Africans, suggesting that the MAF in the hyperlipidaemic participants did not deviate from the majority of general healthy subjects in both Asians and non-Asians. In contrast, in the independent gene candidate studies, the MAF significantly differed (*P* < 0.001 and *P* = 0.006, respectively) than that of neighbouring countries such as Singapore (MAF = 0.445; 21) and Thailand (MAF = 0.542; 20). The variation in the minor allele distribution may be explained by the different sample sizes, therefore resulting in discrepancies in the MAF values.

The presence of minor allele A of the SNP has been associated with decreased CETP concentration, increased HDL-c levels and lower risk of CHD (10, 12) as a result of possible population-dependent variation (13). However, we were unable to demonstrate a difference in HDL-c levels between females and males presumably because the study effect size was too small to detect the association. The SNP had been reported to confer protective effects, for example the minor allele A carriers of the SNP had a higher HDL-c and lower LDL-c level (7, 27, 29). Therefore, the minor allele A carriers are associated with reduced risk of CVD (*P* < 0.001) due to the higher HDL-c level (30, 31). Without taking the SNP factor into account, our findings (Table 1) showed that females had a lower risk of atherogenic dyslipidaemia due to higher HDL-c levels (*P* < 0.001) than males prior to statin treatment. Once statin treatment began (Table 3), and taking the *CETP* rs708272 into account, a similar degree of statin-related TC and LDL-c lowering effect (*P* < 0.001) was observed in both genders, indicating that there was no gender-specific effect on the lipid profiles. Further gender stratification indicated that the *CETP* rs708272 only resulted in significant TG decrease in females with the GG genotype (*P* < 0.001 after 0 month–6 months and *P* = 0.021 after 7 months–12 months), implying that TG levels disproportionately affected females. In contrast, a case-control study (*n* = 640) among Pakistani participants found that the combined effects of four risk SNPs, including rs708272, significantly increased TG level (*r* = 0.127, *P* = 0.001) despite the fact that there was no direct association between the combined risk alleles and CAD (32). Other factors, such as smoking and ethnicity, could explain the contradictory findings in the association of *CETP* rs708272 with the TG levels (21, 33).

In terms of drug effect on lipid parameters, it has been established that the effects of lipid-lowering drugs, including statins, may be influenced by not only genetics, but also other factors, such as gender (21, 34, 35). Therefore, we stratified our analysis based on the participant’s gender. Without taking genetic factors into account, a study conducted among Korean population (*n* = 4,465) demonstrated that female had higher HDL-c level than male (43.8 mg/dL versus 46.3 mg/dL; *P* < 0.001) after adjusting for age factor (35). Consistently, a study conducted across six countries, namely Canada, China, Israel, Poland, Russia and United States (*n* = 19,321), found that the mean HDL-c level in females was also higher (*P* < 0.001) (34). Higher oestrogen levels in females has been proposed as a possible explanation for the findings (35) because the hormone modulates macrophage lipoprotein metabolism, resulting in reduced lipid build-up (36, 37). Aside from hormonal factor (35), the wide range of the HDL-c level disparities between genders could be attributed to ethnicity (34).

We believe that the *CETP* rs708272 did not substantially influence the already established pharmacological effects of statins in terms of LDL-c-lowering effects. Instead, the SNP increased the risk of CVD among minor allele A carriers prior to statin treatment due to the significantly higher LDL levels (*P* = 0.007) (Table 3). In Turkish CHD patients (*n* = 145), the combination of the SNP with statin medication improved anti-atherogenic LDL-1 and large-LDL subfractions (29). Consistently, in the Thai population (*n* = 225), the AA genotype of the SNP was associated with a poorer response in LDL-c reduction after 3 months of simvastatin treatment when compared to GG+GA genotypes (−22.54% versus −35.19%; *P* = 0.028), suggesting that the minor allele A had a higher atherogenic effect (20). Our findings, however, contradicted previously shown hypothesis that the minor allele A exerted an anti-atherogenic effect by reducing CETP activity and lowering LDL-c levels (10, 37) because the minor allele A carriers had significantly higher LDL-c levels (*P* = 0.007) prior to the statin treatment. The anti-atherogenic effect of minor allele A, which resulted in a poorer LDL lowering effects, was attributed to large LDL sub-fractions as previously suggested (38). In fact, Ordovas et al. (13) found that the AA genotype was associated with the highest LDL size (*P* < 0.001), further suggesting that the anti-atherogenic effect was associated with the minor allele A. However, the effect of *CETP* rs708272 on the LDL-c level was different depending on the patient’s condition since minor allele A carriers caused elevation in large LDL sub-fractions in CHD patients only, but not in healthy control group (29). Furthermore, the role of CETP in atherogenicity may be dependent on LDL receptor integrity (7), possibly explaining the increased LDL-c baseline levels observed in the minor allele A female carriers with hyperlipidaemia in this study. Because of the increased LDL-c levels observed, the defective LDL receptor was expected to be more common in females who carried the minor allele A.

For the first time, we were able to describe the MAF of the *CETP* rs708272 for Malaysian Malays (the majority of the participants) as well as whether the SNP was associated with a different lipid profile among statin users. The SNP was found to be associated with lipid profiles, particularly HDL-c, in population-based studies in both non-statin (13, 14), and more recently, statin users (16). However, due to a number of limitations, we were unable to indicate such association in this study. Other confounding factors associated with lipid metabolism, such as BMI, smoking, and alcohol intake (39–40), have to be taken into account. For statistical control of the confounding factors, regression adjustment analyses should be employed (41) with the above-mentioned potential confounders incorporated into the model analysis. Because the participants were chosen at random throughout the enrolment process, the potential selection bias (41) was minimised in this study. The participants in this study were limited to individuals who were hyperlipidaemic and were sampled at a single centre in Peninsular Malaysia’s east coast, which was dominated by Malay ethnicity. Therefore, the findings must be interpreted with caution when replicated in other healthy cohorts or ethnic groups. Given the Malaysia’s multi-ethnic population, future research that includes other ethnic groups could provide a more comprehensive picture of the studied SNP’s impact on Malaysians. Finally, the statin efficacy would be attributed to an additive effect from other genes as well. Proprotein convertase subtilisin/kexin type 9 (PCSK9), which is involved in hepatic uptake of LDL-c and has been linked to a significant reduction in plasma LDL-c levels (42), is another candidate gene that can modulate LDL-c levels. In order to evaluate the potential gene-gene interactions, future research should explore incorporating the impact of other genes such as the PCSK9 gene.

## Conclusion

We conclude that *CETP* rs708272 is associated with different LDL-c and TG, but not HDL-c levels in females and males prior to statin treatment. After statin administration, only the GG genotype in females was associated with lower TG levels whereas the *CETP* rs708272 had no lowering effect on LDL-c and TC in either gender.

## Figures and Tables

**Table 1:** Baseline characteristics of the study population and a stratified analysis by gender group

Participants’ characteristics	All participants (*n* = 229)	Female (*n* = 122 )	Male (*n* = 107)	*P*-value*
Age, mean ± SD (range)	53.0 ± 7.16 (29–69)	53.48 ± 7.20 (29–69)	52.44 ± 7.09 (30–66)	0.274^a^
Race, *n* (%)				
Malay	220 (96.1)	116 (95.1)	104 (97.2)	1.000^b^
Chinese	7 (3.1)	4 (3.3)	3 (2.8)	
Indian	1 (0.4)	1 (0.8)	0 (0.0)	
Others	1 (0.4)	1 (0.8)	0 (0.0)	
*CETP* rs708272, *n* (%)				
GG	89 (38.9)	54 (44.3)	35 (32.7)	0.074^c^
GA + AA	140 (61.1)	68 (55.7)	72 (67.3)	
Statin used, *n* (%)				
Atorvastatin	147 (64.2)	75 (61.5)	72 (67.3)	0.504^b^
Simvastatin	60 (26.2)	35 (28.7)	25 (23.4)	
Pravastatin	16 (7.0)	10 (8.2)	6 (5.6)	
Lovastatin	6 (2.6)	2 (1.6)	4 (3.7)	
Diagnosed clinical manifestation, *n* (%)				
Hypertension with diabetes				
Yes	90 (50.8)	47 (49.5)	43 (52.4)	0.694^c^
No	87 (49.2)	48 (50.5)	39 (47.6)	
Hyperlipidaemia with diabetes				
Yes	29 (55.8)	17 (63.0)	12 (48.0)	0.278^c^
No	23 (44.2)	10 (37.0)	13 (52.0)	
Concurrent drug with statin, *n* (%)				
Anti-hypertensive drugs and diabetic medications	88 (38.4)	43 (35.2)	45 (42.1)	0.715^c^
Anti-hypertensive drugs only	85 (37.1)	49 (40.2)	36 (33.6)	
None	35 (15.3)	19 (15.6)	16 (15.0)	
Diabetic medications only	21 (9.2)	11 (9.0)	10 (9.3)	
Anti-hypertensive drugs, *n* (%)				
Combination of two or more anti-hypertensive drugs	99 (57.2)	56 (60.2)	43 (53.7)	**0.050** b
Calcium channel blockers only	33 (19.1)	22 (23.7)	11 (13.7)	
Angiotensin converting enzyme (ACE) inhibitor only	24 (13.9)	7 (7.5)	17 (21.3)	
Angiotensin receptor blocker only	10 (5.8)	6 (6.4)	4 (5.0)	
Diuretic drugs only	4 (2.3)	1 (1.1)	3 (3.8)	
Beta-blockers only	3 (1.7)	1 (1.1)	2 (2.5)	
Mean lipid level at the baseline (mmol/L)				
TC, mean ± SD (normal range < 5.2)	5.72 ± 1.21	5.85 ± 1.20	5.58 ± 1.21	0.091^d^
HDL-c, mean ± SD (normal range > 1.5)	1.30 ± 0.47	1.36 ± 0.30	1.23 ± 0.60	**< 0.001** a
LDL-c, mean ± SD (normal range < 2.6)	3.72 ± 1.19	3.77 ± 1.25	3.67 ± 1.14	0.538^d^
TG, mean ± SD (normal range < 1.7)	1.65 ± 0.83	1.68 ± 0.82	1.62 ± 0.85	0.481^a^

Notes:

a The Mann-Whitney test was used to compare mean values between females and males;

b Frequencies for the given categories between female and male were compared using Fischer’s exact test;

c Frequencies for the given categories between female and male were compared using Pearson chi-square test;

d The *t*-test was used to compare mean values between females and males;

* *P* ≤ 0.05 is considered statistically significant;

TC = total cholesterol; HDL-c = high density lipoprotein; LDL-c = low density lipoprotein; TG = triglyceride; SD = standard deviation

**Table 2:** MAF values and the analysis for the genotype frequencies of the *CETP* rs708272 in the present study and other populations

Participants	Genotypic frequency, *n* (%)			MAF value	*P*-value

	GG	GA	AA		
Present study (all participants)	89 (38.9)	101 (44.1)	39 (17.0)	0.391	0.736^a^
Southeast Asians					
Thailand (16)	101 (48.8)	97 (46.9)	9 (4.3)	0.278	0.037 b
Thailand (20)	9 (4.0)	188 (83.6)	28 (12.4)	0.542	< 0.001^b^
Singapore (21)	473 (29.8)	813 (51.3)	300 (18.9)	0.445	0.006^b^
East Asian	200 (36.9)	230 (45.6)	74 (14.7)	0.375	0.834^b^
China	46 (49.5)	34 (36.6)	13 (14.0)	0.323	0.081^b^
China (17)	343 (35.4)	442 (45.6)	185 (19.0)	0.419	0.321^b^
China (22)	163 (28.5)	282 (49.4)	126 (22.1)	0.467	0.005^b^
China (23)	70 (35.0)	94 (47.0)	36 (18.0)	0.415	0.408^b^
China (24)	326 (43.0)	336 (44.3)	96 (12.7)	0.348	0.266^b^
Japan	42 (40.4)	50 (48.1)	12 (11.5)	0.356	0.792^b^
Japan (25)	1168 (35.7)	1566 (47.9)	534 (16.4)	0.403	0.341^b^
Other continents					
American	107 (30.8)	159 (45.8)	81 (23.4)	0.463	0.047^b^
European	159 (31.6)	260 (51.7)	84 (16.7)	0.425	0.173^b^
African	374 (56.6)	248 (37.5)	39 (5.9)	0.247	<0.001^b^

Notes:

a Chi-square goodness of fit test was performed to confirm the validity of the observed genotypic frequencies in the present study to the expected frequencies under Hardy-Weinberg equilibrium;

b Pearson chi-square test was used to compare frequencies of GG and GA+AA genotypes between the present study and the indicated population. Data for the MAF was obtained from the 1000 Genomes Project Phase 3 in the Ensembl genome browser (https://asia.ensembl.org/index.html), except for the indicated populations with references;

* *P* ≤ 0.05 is considered as statistically significant;

MAF = minor allele frequency

**Table 3:** Analysis between lipid parameters and *CETP* rs708272 polymorphism in the overall participants and each gender group

Lipid profiles (mmol/L), mean ± SD		Whole sample (*N* = 229)			Female (*N* = 122)			Male (*N* = 107)	
			
		GG (*n* = 89)	GA+AA (*n* = 140)	*P*-value	GG (*n* = 54)	GA+AA (*n* = 68)	*P*-value	GG (*n* = 35)	GA+AA (*n* =72)	*P*-value
TC level									
Baseline level		5.59 ± 1.00	5.81 ± 1.32	0.154^a^	5.62 ± 0.92	6.03 ± 1.35	0.054^a^	5.54 ± 1.12	5.60 ± 1.26	0.798^a^
0–6 months treatment		4.66 ± 0.85	4.95 ± 1.16	0.179^b^	4.71 ± 0.75	5.06 ± 1.34	0.182^a^	4.58 ± 1.01	4.87 ± 1.00	0.301^a^
7–12 months treatment		4.90 ± 0.98	4.90 ± 1.13	0.577^b^	5.17 ± 0.85	5.03 ± 1.17	0.472^a^	4.48 ± 1.04	4.77 ± 1.09	0.211^a^
*P*-value		**< 0.001** c	**< 0.001** d		**< 0.001** d	**< 0.001** d		**0.005** d	**< 0.001** d	
*P*1		**< 0.001**	**< 0.001**		**< 0.001**	**< 0.001**		**0.017**	**0.001**	
*P*2		**0.0**01	**< 0.001**		**< 0.001**	**< 0.001**		**0.031**	**< 0.001**	
*P*3		0.286	**< 0.001**		0.122	1.000		1.000	1.000	
HDL-c level									
Baseline level		1.26 ± 0.26	1.32 ± 0.56	0.612^b^	1.32 ± 0.28	1.39 ± 0.31	0.423^b^	1.18 ± 0.21	1.26 ± 0.72	0.710^b^
0–6 months treatment		1.22 ± 0.24	1.21 ± 0.24	0.943^b^	1.27 ± 0.25	1.28 ± 0.21	0.822^a^	1.12 ± 0.21	1.15 ± 0.25	0.680^a^
7–12 months treatment		1.27 ± 0.30	1.27 ± 0.28	0.912^b^	1.34 ± 0.30	1.36 ± 0.29	0.472^b^	1.15 ± 0.27	1.17 ± 0.22	0.601^b^
*P*-value		0.961^c^	0.457^d^		0.775^c^	0.734^d^		0.554^c^	0.586^d^	
*P*1		–	–		–	–		–	–	
*P*2		–	–		–	–		–	–	
*P*3		–	–		–	–		–	–	
LDL-c level									
Baseline level		3.52 ± 0.95	3.85 ± 1.31	**0.032** a	3.44 ± 0.84	4.02 ± 1.44	**0.007** a	3.64 ± 1.09	3.69 ± 1.17	0.836^a^
0–6 months treatment		2.77 ± 0.78	3.02 ± 0.97	0.179^b^	2.78 ± 0.75	3.13 ± 1.21	0.146^a^	2.75 ± 0.85	2.93 ±0.74	0.423^a^
7–12 months treatment		2.91 ± 0.90	2.95 ± 0.93	0.579^b^	3.07 ± 0.86	2.99 ± 0.95	0.646^a^	2.65 ± 0.91	2.92 ± 0.92	0.056^b^
*P*-value		**< 0.001** d	**< 0.001** d		**< 0.001** d	**< 0.001** d		**0.050** c	**0.006** c	
*P*1		**< 0.001**	**< 0.001**		**< 0.001**	**< 0.001**		0.102	**0.025**	
*P*2		**< 0.001**	**< 0.001**		**0.001**	**< 0.001**		0.102	**0**.0**12**	
*P*3		0.306	1.000		0.117	1.000		1.000	1.000	
TG level									
Baseline level		1.77 ± 0.87	1.58 ± 0.81	0.052^b^	1.90 ± 0.98	1.52 ± 0.63	**0.044** b	1.58 ± 0.64	1.64 ± 0.95	0.484^a^
0–6 months treatment		1.64 ± 0.95	1.65 ± 0.93	0.936^b^	1.66 ± 1.07	1.44 ± 0.56	0.890^b^	1.61 ± 0.74	1.80 ± 1.11	0.826^a^
7–12 months treatment		1.61 ±0.83	1.51 ± 0.88	0.290^b^	1.70 ± 0.85	1.52 ± 0.95	0.157^b^	1.47 ± 0.80	1.50 ± 0.81	0.836^a^
*P*-value		**0.001** c	0.566^c^		**< 0.001** c	0.703^c^		0.846^d^	0.134^c^	
*P*1		**0.001**	–		**< 0.001**	–		–	–	
*P*2		**0.043**	–		**0.021**	–		–	–	
*P*3		0.692	–		0.793	–		–	–	

Notes:

a *P*-values derived from an independent *t*-test;

b *P*-value derived from Mann-Whitney U test;

c *P*-value derived from Friedman’s test for nonparametric variables;

d *P*-value derived from one-way repeated measures ANOVA.

Bonferroni adjustment were performed for post-hoc analysis and the *P*-values are indicated by *P*1 (baseline versus 0–6 months treatment), *P*2 (Baseline versus 7–12 months treatment) and *P*3 (0–6 months versus 7–12 months treatment); *P* ≤ 0.05 is considered as statistically significant (numbers are bold); *P*1 = comparison of baseline lipid levels versus 0–6 months after statin treatment, *P*2 = comparison of baseline lipid levels versus 7–12 months after statin treatment, *P*3 = comparison of lipid levels between 0–6 months and 7–12 months after statin treatment; TC = total cholesterol; HDL-c = high density lipoprotein; LDL-c = low density lipoprotein; TG = triglyceride

**Figure:**
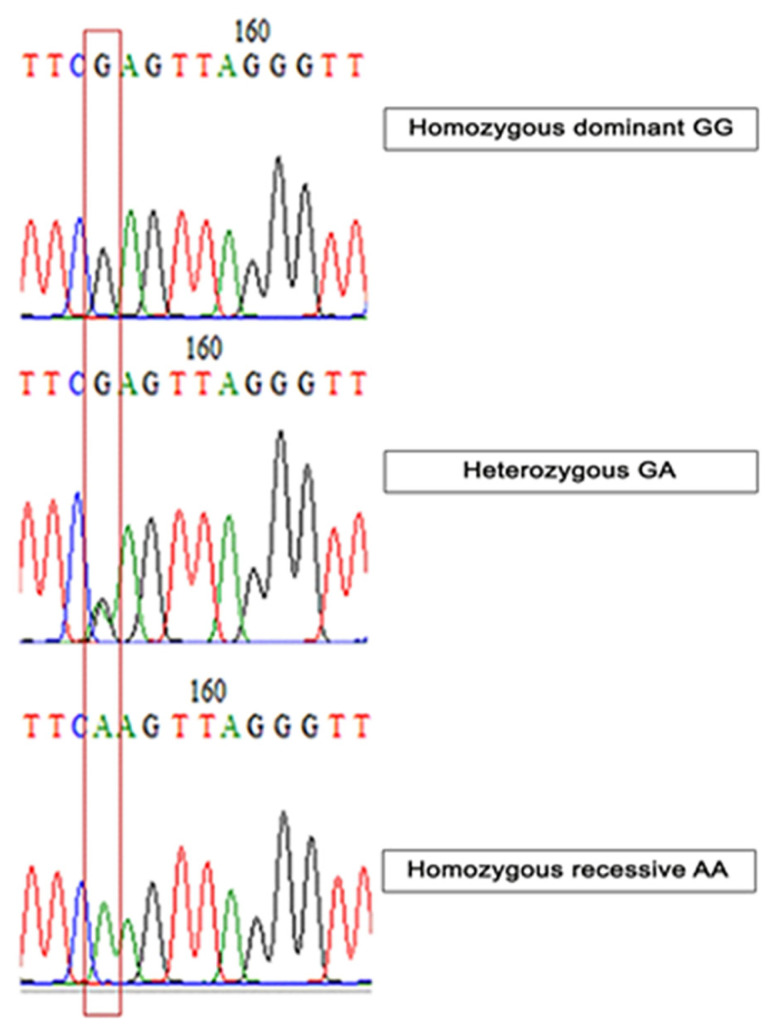
Chromatogram indicates the DNA sequence of homozygous dominant GG, heterozygous GA and homozygous recessive AA genotypes of the *CETP* rs708272

## References

[1] Endo A, Tsujita Y, Kuroda M, Tanzawa K (1977). Inhibition of cholesterol synthesis in vitro and in vivo by ML-236A and ML-236B, competitive inhibitors of 3-hydroxy-3-methylglutaryl-coenzyme A reductase. Eur J Biochem.

[2] Schaiff RAB, Moe RM, Krichbaum DW (2008). An overview of cholesterol management. Am Heal Drug Benefits.

[3] Baigent C, Keech A, Kearney P, Blackwell I, Buck G, Pollicino C (2005). Efficacy and safety of cholesterol-lowering treatment: prospective meta-analysis of data from 90 056 participants in 14 randomised trials of statins. Lancet.

[4] Vladimirova-Kitova LG, Kitov SI, Sekar Ashok K (2015). Resistance of statin therapy, and methods for its influence. Hypercholesterolemia.

[5] Yen FT, Deckelbaum RJ, Mann CJ, Marcel YL, Milne RW, Tall AR (1989). Inhibition of cholesteryl ester transfer protein activity by monoclonal antibody. Effects of cholesteryl ester formation and neutral lipid mass transfer in human plasma. J Clin Invest.

[6] Hannuksela ML, Johanna Liinamaa M, Antero Kesaniemi Y, Savolainen MJ (1994). Relation of polymorphisms in the cholesteryl ester transfer protein gene to transfer protein activity and plasma lipoprotein levels in alcohol drinkers. Atherosclerosis.

[7] Oliveira HCF, De Faria EC (2011). Cholesteryl ester transfer protein: the controversial relation to atherosclerosis and emerging new biological roles. IUBMB Life.

[8] Radovica I, Fridmanis D, Vaivade I, Nikitina-Zake L, Klovins J (2013). The association of common SNPs and haplotypes in CETP gene with HDL cholesterol levels in Latvian population. PLoS ONE.

[9] Deek R, Nasser J, Ghanem A, Mardelli M, Khazen G, Salloum AK (2019). Genome-wide association analysis of HDL-C in a Lebanese cohort. PLoS ONE.

[10] Semaev S, Shakhtshneider E, Orlov P, Ivanoshchuk D, Malyutina S, Gafarov V (2019). Association of rs708272 (CETP gene variant) with lipid profile parameters and the risk of myocardial infarction in the white population of western Siberia. Biomolecules.

[11] Drayna D, Lawn R (1987). Multiple RFLPs at the human cholesteryl ester transfer protein (CETP) locus. Nucleic Acids Res.

[12] Nagano M, Yamashita S, Hirano KI, Takano M, Maruyama T, Ishihara M (2004). Molecular mechanisms of cholesteryl ester transfer protein deficiency in Japanese. J Atheroscler Thromb.

[13] Ordovas JM, Cupples LA, Corella D, Otvos JD, Osgood D, Martinez A (2000). Association of cholesteryl ester transfer protein-TaqIB polymorphism with variations in lipoprotein subclasses and coronary heart disease risk: The framingham study. Arterioscler Thromb Vasc Biol.

[14] Thompson A, Di Angelantonio E, Sarwar N, Erqou S, Saleheen D, Dullaart RPF (2008). Association of cholesteryl ester transfer protein genotypes with CETP mass and activity, lipid levels, and coronary risk. J Am Med Assoc.

[15] Schierer A, Been LF, Ralhan S, Wander GS, Aston CE, Sanghera DK (2012). Genetic variation in cholesterol ester transfer protein, serum CETP activity, and coronary artery disease risk in Asian Indian diabetic cohort. Pharmacogenet Genomics.

[16] Srisawasdi P, Rodcharoen P, Vanavanan S, Chittamma A, Sukasem C, Nakorn CN (2021). Association of CETP gene variants with atherogenic dyslipidemia among thai patients treated with statin. Pharmgenomics Pers Med.

[17] Cai G, Shi G, Huang Z (2018). Gender specific effect of CETP rs708272 polymorphism on lipid and atherogenic index of plasma levels but not on the risk of coronary artery disease: a case-control study. Medicine.

[18] Kauma H, Savolainen MJ, Heikkilä R, Rantala AO, Lilja M, Reunanen A (1996). Sex difference in the regulation of plasma high density lipoprotein cholesterol by genetic and environmental factors. Hum Genet.

[19] Lemeshow S, Hosmer DW, Klar J, Lwanga SK (1990). Adequacy of sample size in health studies.

[20] Wanmasae S, Sirintronsopon W, Porntadavity S, Jeenduang N (2017). The effect of APOE, CETP, and PCSK9 polymorphisms on simvastatin response in Thai hypercholesterolemic patients. Cardiovasc Ther.

[21] Lu Y, Tayebi N, Li H, Saha N, Yang H, Heng CK (2013). Association of CETP Taq1B and −629C > A polymorphisms with coronary artery disease and lipid levels in the multi-ethnic Singaporean population. Lipids Health Dis.

[22] Hou H, Ma R, Guo H, He J, Hu Y, Mu L (2017). Association between six CETP polymorphisms and metabolic syndrome in uyghur adults from Xinjiang, China. Int J Environ Res Public Health.

[23] Yue YH, Bai XD, Zhang HJ, Li YM, Hu L, Liu LY (2016). Gene polymorphisms affect the effectiveness of atorvastatin in treating ischemic stroke patients. Cell Physiol Biochem.

[24] Zhou Y, Yin R, Deng Y, Li Y, Wu J (2008). Interactions between alcohol intake and the polymorphism of rs708272 on serum high-density lipoprotein cholesterol levels in the Guangxi Hei Yi Zhuang population. Alcohol.

[25] Hishida A, Wakai K, Naito M, Suma S, Sasakabe T, Hamajima N (2014). Polymorphisms of genes involved in lipid metabolism and risk of chronic kidney disease in Japanese-Cross-sectional data from the J-MICC study. Lipids Health Dis.

[26] Mabuchi H, Nohara A, Inazu A (2014). Cholesteryl ester transfer protein (CETP) deficiency and CETP inhibitors. Mol Cells.

[27] Ruiz-Iruela C, Candás-Estébanez B, Pintó-Sala X, Baena-Díez N, Caixàs-Pedragós A, Güell-Miró R (2020). Genetic contribution to lipid target achievement with statin therapy: a prospective study. Pharmacogenomics J.

[28] Chu WC, Aziz AFA, Nordin AJ, Cheah YK (2016). Association of cholesteryl ester transfer protein and endothelial nitric oxide synthase gene polymorphisms with coronary artery disease in the multi-ethnic Malaysian population. Clin Appl Thromb.

[29] Kanca D, Gormus U, Tokat B (2016). Additive antiatherogenic effects of CETP rs708272 on serum LDL subfraction levels in patients with CHD under statin therapy. Biochem Genet.

[30] Guo SX, Yao MH, Ding YS, Zhang JY, Yan YZ, Liu JM (2016). Associations of cholesteryl ester transfer protein TaqIB polymorphism with the composite ischemic cardiovascular disease risk and HDL-C concentrations: A meta-analysis. Int J Environ Res Public Health.

[31] Li YY, Wu XY, Xu J, Qian Y, Zhou CW, Wang B (2013). Apo A5-1131T/C, FgB-455G/A, −148C/T, and CETP TaqIB gene polymorphisms and coronary artery disease in the Chinese population: a meta-analysis of 15,055 subjects. Mol Biol Rep.

[32] Shahid SU, Shabana NA, Cooper JA, Rehman A, Humphries SE (2017). Common variants in the genes of triglyceride and HDL-C metabolism lack association with coronary artery disease in the Pakistani subjects. Lipids Health Dis.

[33] Yilmaz H, Isbir T, Agachan B, Karaali ZE (2005). Effects of cholesterol ester transfer protein Taq1B gene polymorphism on serum lipoprotein levels in Turkish coronary artery disease patients. Cell Biochem Funct.

[34] Davis CE, Williams DH, Oganov RG, Tao S, Rywik SL, Stein Y (1996). Sex difference in high density lipoprotein cholesterol in six countries. Am J Epidemiol.

[35] Kim HJ, Park HA, Cho YG, Kang JH, Kim KW, Kang JH (2011). Gender difference in the level of HDL cholesterol in Korean adults. Korean J Fam Med.

[36] McCrohon JA, Nakhla S, Jessup W, Stanley KK, Celermajer DS (1999). Estrogen and progesterone reduce lipid accumulation in human monocyte-derived macrophages: A sex-specific effect. Circulation.

[37] Barter PJ, Brewer HB, Chapman MJ, Hennekens CH, Rader DJ, Tall AR (2003). Cholesteryl ester transfer protein: a novel target for raising HDL and inhibiting atherosclerosis. Arterioscler Thromb Vasc Biol.

[38] Packard C, Caslake M, Shepherd J (2000). The role of small, dense low density lipoprotein (LDL): a new look. Int J Cardiol.

[39] Denke MA, Sempos CT, Grundy SM (1993). Excess body weight. An underrecognized contributor to high blood cholesterol levels in white American men. Arch Intern Med.

[40] Zhang L, He S, Li Z, Gan X, Li S, Cheng X (2019). Apolipoprotein E polymorphisms contribute to statin response in Chinese ASCVD patients with dyslipidemia. Lipids Heal Dis.

[41] Haneuse S (2016). Distinguishing selection bias and confounding bias in comparative effectiveness research. Med Care.

[42] Sahebkar A, Watts GF (2013). New LDL-cholesterol lowering therapies: pharmacology, clinical trials, and relevance to acute coronary syndromes. Clin Ther.

